# Plutonium, ^90^Sr and ^241^Am in human bones from southern and northeastern parts of Poland

**DOI:** 10.1007/s10967-013-2850-y

**Published:** 2013-11-27

**Authors:** Kamil Brudecki, Jerzy W. Mietelski, Robert Anczkiewicz, Edward B. Golec, Ewa Tomankiewicz, Konstanty Kuźma, Paweł Zagrodzki, Joanna Golec, Sebastian Nowak, Elżbieta Szczygieł, Zbigniew Dudkiewicz

**Affiliations:** 1 Institute of Nuclear Physics, Polish Academy of Sciences, Kraków, Poland; 2 Institute of Geological Sciences, Polish Academy of Sciences, Kraków, Poland; 3 5th Military Clinical Hospital with Polyclinic, Kraków, Poland; 4 Faculty of Motor of the Bronislaw Czech University School of Physical Education, Kraków, Poland; 5 Faculty of Physical Therapy, Administration College, Bielsko-Biała, Poland; 6 General Hospital in Bielsk Podlaski, Bielsk Podlaski, Poland; 7 Chair of Bromathology, Jagiellonian University Medical College, Kraków, Poland; 8 Hand Surgery Clinic, Chair of Traumatology and Orthopaedics, Medical University Łódź, Lodz, Poland

**Keywords:** Plutonium, Americium, Strontium, Chernobyl, Mass spectrometry, Pu isotopic ratios, Human bones

## Abstract

The paper presents the results of our study on ^238^Pu, ^239^Pu, ^240^Pu, ^241^Am and ^90^Sr concentration in human bones carried out on a set of 88 individual samples of central Europe origin. Bone tissue samples were retrieved under surgery while introducing hip joint implants. The conducted surgeries tend to cover either southern or northeastern parts of Poland. While for the southern samples only global fallout was expected to be seen, a mixed global and Chernobyl fallout were to be reflected in the others. Alpha spectrometry was applied to obtain activity concentration for ^238^Pu, ^239+240^Pu, ^241^Am, while liquid scintillation spectrometry for ^90^Sr and mass spectrometry to receive ^240^Pu/^239^Pu mass ratio. Surprisingly enough, and to the contrary to our expectations we could not see any significant differences in either Pu activity or Pu mass ratio between the studied populations. In both populations Chernobyl fraction proved marginal. The results on ^90^Sr and ^241^Am confirm similarities between the two examined groups.

## Introduction

**Fig. 1:**
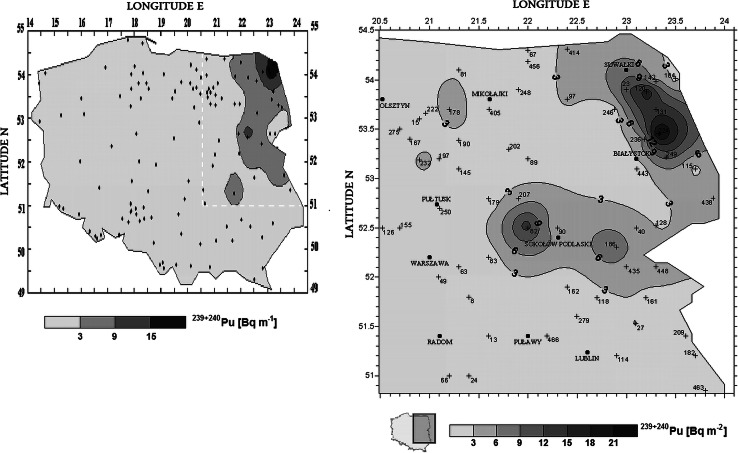
Approximate map of ^239+240^Pu deposition from Chernobyl fallout in Poland left: first approximation [6] et al. right result of more detailed study [7]

Plutonium isotopes present in Polish environment are of two sources. The first is Global Fallout (GF), a radioactive contamination formed as a result of nuclear weapons tests carried out in the last century. Among the effects of such tests, a spread in atmosphere of about 4.5 t of ^239^Pu, 1 t of ^240^Pu, 200 kg of ^242^Pu, 95 kg of ^241^Pu and 1 kg of ^238^Pu was observed [1]. As the result, for example, the average annual air concentration of ^239+240^Pu in Finland in 1963 reached 20,000 nBq m^−3^ [2]. At present, Pu in air concentration falls usually below 10 nBq m^−3^ [3]. The second source of Pu in air is Chernobyl. Due to the reactor explosion on the night of 25 April 1986 (23:23 GMT, or 1:23 on 26 April in Moscow time) Pu in the amounts much smaller than in GF were released to the atmosphere. Namely, 14 kg of ^239^Pu, 4 kg of ^240^Pu, 48 g of ^238^Pu and 1.4 kg of ^241^Pu [4]. Most of this mass settled near the damaged reactor, but some of them migrated over long distances. Just after the accident a radioactive cloud migrated onto the northern-east part of Poland, contaminating this region with Chernobyl ^239+240^Pu, deposited in a form of small hot particles [5]. The maximum of ^239+240^Pu deposition from Chernobyl within this region reached 24 Bqm^−2^. Geographical distribution of plutonium contamination is shown in Fig. 1 [6, 7]. Much higher Pu cumulated deposition, even slightly exceeding 200 Bqm^−2^ for ^239+240^Pu, were noticed in High Tatra Mountains in the southern Poland, though it was pure global fallout [8]. The average deposition of ^239+240^Pu for the latitude belt of Poland from GF is believed to be 58 Bqm^−2^ [9].

The main exposure pathway for plutonium is inhalation. Plutonium deposition in the respiratory system mainly depends on aerosols diameter, individual’s age, sex, and lifestyle, reaching from 11 to 99 % of the inhaled plutonium from the air [10, 11]. Subsequently, a part of plutonium is transferred from the respiratory to gastro–intestine system, where almost all Pu is excreted (f_1_ for plutonium is only 0.0005) and the rest is transferred to blood. Plutonium from blood is accumulated mainly in liver and bones, where it remains for many years [12].

The study has been driven by our search to estimate and compare plutonium internal contamination in humans living in southern and north-eastern parts of Poland. For the southern Poland we expected to find only global fallout tracers, while for northeastern Poland a mixed global and Chernobyl fallout was likely to be detected. Besides plutonium isotopes, we estimated also internal contamination with ^241^Am and ^90^Sr.

## Material and method

In our study bone tissue samples obtained during routine surgeries, namely implanting hip joint replacements, were studied. Patients of general population, were in no special way exposed to radioactive contamination. Surgeries were conducted at the 5th Military Hospital in Kraków (in Southern Poland) and at the General Hospital of Bielsk Podlaski (Northeastern Poland). As the patients originated broadly from the vicinity of the hospitals, Pu only from GF was expected for Kraków, while mixed GF and Chernobyl fallout was expected for Bielsk Podlaski. Both hospital studies were granted approval by the appropriate council for ethics in medical research.

To extract plutonium isotopes we applied the method described in details elsewhere [13–15]. The samples were ashed at 600 °C for 2 days. Tracers such as ^242^Pu, ^243^Am and ^85^Sr were added. The ashes were gently soaked with 6 M HCl, then heated, evaporated to nearly dryness. Hydrogen peroxide was added to each sample to destroy carbon traces. Next, the samples were dissolved in 1 M HNO_3_. For each sample precipitation of calcium oxalates at pH = 3 was completed to obtain the transuranic (Pu + Am) fraction. Calcium oxalates were dissolved with 1 M HNO_3_. The oxidation state of Pu was adjusted to +4 by means of hydrazine and NaNO_2_ [16], and after conversion into 8 M HNO_3_ the fraction was passed through Dowex-1 × 8 column. Th was washed out with 12 M HCl. Pu retained on the column was eluted with 50 mL of 0.1 M HF–0.1 M HCl. Alpha spectrometric Pu sources were prepared directly in the solutions by means of NdF_3_ method [17]. Pu sources were measured with Silena AlphaQuattro spectrometer equipped with four Canberra PIPS detectors. A typical measurement time was about 600 000 s. We also took the opportunity to extract and measure ^241^Am and ^90^Sr following procedures described previously [13].

Later, all alpha spectrometric plutonium sources were dissolved and prepared for measurement on the mass spectrometry. The NdF_3_ source with a plastic filter was dissolved in a solution of concentrated HCl and HNO_3_ with a dish of boric acid added. The solution was evaporated several times to reach near dryness. To make sure that the source was dissolved, we used also HClO_4_ [18]. Then, we purified the samples from uranium and thorium tracers. The Pu oxidation state was again adjusted to +4 (as described above) and converted into 4 M HNO_3_. The solutions were passed through Triskem International TEVA Resin. This was performed to remove traces of uranium, which passes the column without retention. Thorium, which together with Pu remains in the column, was eluted with the concentrated HCl and then Pu was eluted with 15 mL of 0.1 M HF–0.1 M HCl. The last step was to converse samples into 300 μL of 2 % HNO_3_—0.1 % HF and to measure with mass spectrometer MC ICPMS Neptune by Thermoelectron Finningan.

Along with the samples two reference material (RM) were analysed. The first one, NIST 4356 [19] was used to check alpha spectrometry results. Ashed animal bones provided the matrix for this reference material. In the course of the study, we measured RM four times. The comparison between the received and the reference values are presented in Table 1. The second RM was NIST 947 [20] and we used it to check mass spectrometer results. Over the 4 days when mass spectrometer was used the second RM material was measured 8 times. The comparison between the received and reference values are presented in Table 2.

**Table 1:** Comparison between the received and reference values for the standard reference material NIST 4356 [19]

Isotopes	Sample number	Received value	Certified value	Tolerance limit
^238^Pu (mBq g^−1^)	1	0.82 ± 0.12	0.86 ± 0.01	0.73–1.00
	2	0.84 ± 0.12		
	3	0.88 ± 0.07		
	4	0.90 ± 0.10		
^239+240^Pu (mBq g^−1^)	1	1.10 ± 0.13	1.26 ± 0.03	1.09–1.65
	2	1.10 ± 0.12		
	3	1.24 ± 0.09		
	4	1.14 ± 0.12		
^241^Am (mBq g^−1^)	1	12.2 ± 0.7	9.98	8.76–13.6
	2	10.6 ± 1.1		
	3	10.5 ± 1.4		
	4	10.2 ± 0.9		
^90^Sr (mBq g^−1^)	1	41 ± 9	42.6 ± 0.9	36.4–49.5
	2	–		
	3	37 ± 3		
	4	40 ± 2

**Table 2:** Comparison between the received and reference values for the standard reference material NIST 947 [20]

Day	Received value	Certified value
04.08.2011	0.241 ± 0.004	0.241
	0.240 ± 0.005	
06.09.2011	0.249 ± 0.001	
07.09.2011	0.251 ± 0.001	
	0.250 ± 0.001	
08.09.2011	0.246 ± 0.002	
	0.253 ± 0.001	
	0.244 ± 0.001	

The difference between the characteristic plutonium mass ratios (^240^Pu to ^239^Pu) in the global and Chernobyl fallout allowed us to distinguish the two. By solving the equation system given below

1 $$ \frac{{_{G}^{240} N}}{{_{G}^{239} N}} = \alpha $$

2 $$ \frac{{_{Ch}^{240} N}}{{_{Ch}^{239} N}} = \beta $$

where

^240^*N*: stands for the observed number of counts ^240^Pu
^239^*N*: stands for the observed number of counts ^239^Pu
$$ _{G}^{240} N $$
stands for the observed number of counts ^240^Pu, come from global fallout
$$ _{G}^{239} N $$
stands for the observed number of counts ^239^Pu, come from global fallout
$$ _{Ch}^{240} N $$
stands for the observed number of counts ^240^Pu, come from Chernobyl fallout
$$ _{Ch}^{239} N $$
stands for the observed number of counts ^239^Pu, come from Chernobyl fallout
α: is the model ratio, characteristic for the global fallout (assumed 0.18 [21])
β: is the model ratio, characteristic for Chernobyl fallout (assumed 0.40 [21])

we can prove that the percent of Chernobyl fraction F can be expressed as

3 $$ F = \left( {\frac{{^{240} N}}{{^{239} N}} - \alpha } \right)*\left( {\beta - \alpha } \right)^{ - 1}\,*\,100\,\% $$

## Results

In total we analysed bone samples of 88 patients. The group of Southern Poland inhabitants contained 60 people, 20 men, 37 women, and 3 person whose gender had not been specified in the questionnaire. The youngest patient was 41 years old, while the oldest 88. For Northeastern Poland bones 28 people were involved, 11 of them were men and 17 women. Their age ranged between 32 and 90. The obtained concentration ranges for the studied isotopes in booth populations are presented in Table 3. In all the samples ^238^Pu activity was found to be below the minimum detection activity concentration, that is on the level of a single mBqkg^−1^. The completed results for booth group are presented in Table 4. It is noticeable that the data presented in Tables 3 and 4 do not show any significant differences for the studied isotopes concentration in bones between the members of both groups.

**Table 3:** Obtained activity ranged of studied isotopes in both studied populations

Population	Isotopes	Min	Max	Average	Median
Kraków (Southern Poland)	^239+240^Pu (mBq kg^−1^)	<2	42 ± 12	15 ± 9	14 ± 3
	^241^Am (mBq kg^−1^)	<2	35 ± 12	12 ± 7	11 ± 2
	^90^Sr (Bq kg^−1^)	1.6 ± 0.3	18 ± 1	6.5 ± 4.1	5.2 ± 0.2
Bielsk Podlaski (North-eastern Poland)	^239+240^Pu (mBq kg^−1^)	<2	50 ± 4	16 ± 10	14 ± 3
	^241^Am (mBq kg^−1^)	<2	20 ± 4	7 ± 5	6 ± 3
	^90^Sr (Bq kg^−1^)	1.8 ± 0.4	19 ± 2	6.4 ± 3.9	5.2 ± 3.8

**Table 4:** Obtained activity for each individual of the studied isotopes in both populations under study

Code of individual KR—Kraków (Southern Poland) BP—Bielsk Podlaski (Northereastern Poland)	Age	Sex	^238^Pu (mBq kg^−1^)	^239+240^Pu (mBq kg^−1^)	^241^Am (mBq kg^−1^)	^90^Sr (Bq kg^−1^)	Mass ratio ^240^Pu/^239^Pu	F (%)
KR1	56	M	<2	6.1 ± 1.1	7.1 ± 2.1	9.3 ± 0.9	0.0839 ± 0.0006	–
KR2	76	M	<3	20.1 ± 2.3	11.1 ± 2.6	12.4 ± 1.5	0.1912 ± 0.0011	5.1 ± 0.5
KR3	59	M	<3	15.3 ± 2.7	4.7 ± 1.6	12.9 ± 1.3	–	–
KR4	76	F	<4	15.5 ± 2.4	7.3 ± 3.5	4.2 ± 0.4	0.1161 ± 0.0008	–
KR5	78	F	<4	7.5 ± 1.6	7.2 ± 3.6	6.2 ± 0.7	0.0983 ± 0.0012	–
KR6	59	M	<3	5.7 ± 1.1	5.5 ± 2.3	3.6 ± 0.5	0.1818 ± 0.0020	0.8 ± 0.9
KR7	–	–	<5	16.1 ± 2.7	2.8 ± 2.4	3.3 ± 0.4	0.1661 ± 0.0014	–
KR8	–	–	<3	22.8 ± 6.2	7.1 ± 4.9	1.9 ± 0.2	0.1925 ± 0.0013	5.7 ± 0.6
KR9	–	–	<2	17.2 ± 2.0	7.3 ± 1.4	13.4 ± 1.9	0.1997 ± 0.0017	9.0 ± 0.8
KR10	64	F	<3	11.9 ± 3.9	23.0 ± 12.0	10.9 ± 0.9	0.1921 ± 0.0017	5.5 ± 0.8
KR11	73	F	<5	16.4 ± 2.7	8.9 ± 3.4	4.7 ± 0.6	0.1902 ± 0.0011	4.6 ± 0.5
KR12	88	F	<10	17.3 ± 3.2	23.0 ± 7.7	1.6 ± 0.3	0.1863 ± 0.0015	2.9 ± 0.7
KR13	62	F	<4	9.5 ± 1.9	3.6 ± 3.1	9.4 ± 1.3	0.1878 ± 0.0019	3.5 ± 0.9
KR14	84	M	<5	14.0 ± 2.6	15.0 ± 12.0	7.7 ± 1.1	0.1842 ± 0.0021	1.9 ± 1.0
KR15	64	M	<2	27.1 ± 5.4	11.4 ± 4.8	8.9 ± 0.7	0.1869 ± 0.0006	3.1 ± 0.3
KR16	63	M	<2	15.7 ± 4.9	5.8 ± 2.1	13.5 ± 1.2	0.1938 ± 0.0010	6.3 ± 0.5
KR17	60	M	<3	10.8 ± 5.6	16.0 ± 4.7	4.9 ± 1.3	0.1964 ± 0.0012	7.5 ± 0.5
KR18	77	F	<6	16.0 ± 2.9	19.8 ± 4.7	7.4 ± 0.9	0.1899 ± 0.0018	4.5 ± 0.8
KR19	76	F	<5	13.7 ± 2.4	8.6 ± 3.9	3.6 ± 0.4	0.1844 ± 0.0010	2.0 ± 0.5
KR 20	75	F	<4	14.0 ± 2.2	7.6 ± 3.9	15.0 ± 2.0	0.1912 ± 0.0017	5.1 ± 0.8
KR 21	73	F	<3	9.3 ± 1.7	2.7 ± 4.7	6.0 ± 0.6	0.1929 ± 0.0015	5.9 ± 0.7
KR 22	71	F	<7	10.4 ± 2.3	12.6 ± 8.8	2.0 ± 0.6	0.1839 ± 0.0030	1.8 ± 1.4
KR 23	78	M	<3	25.0 ± 3.2	20.4 ± 6.7	5.4 ± 0.6	0.1961 ± 0.0011	7.3 ± 0.5
KR 24	42	M	<1	<2	<2	11.9 ± 2.8	0.1291 ± 0.0017	–
KR 25	66	M	<1	24.9 ± 1.9	8.3 ± 2.2	2.7 ± 0.3	0.1899 ± 0.0018	4.5 ± 0.8
KR 26	53	M	<2	12.4 ± 1.7	7.0 ± 2.1	9.9 ± 1.2	0.1876 ± 0.0017	3.5 ± 0.8
KR 27	70	F	<2	15.1 ± 2.4	10.0 ± 3.3	9.7 ± 0.7	0.1923 ± 0.0017	5.6 ± 0.8
KR 28	73	F	<3	12.6 ± 2.2	10.4 ± 3.1	11.6 ± 0.5	0.1910 ± 0.0015	5.0 ± 0.7
KR 29	63	M	<1	16.4 ± 1.9	9.4 ± 1.7	1.9 ± 0.1	0.2022 ± 0.0017	10.1 ± 0.8
KR 30	78	M	<4	15.0 ± 3.0	18.2 ± 5.2	7.5 ± 0.3	0.1916 ± 0.0018	5.3 ± 0.8
KR 31	82	F	<5	17.7 ± 4.4	11.3 ± 6.3	4.2 ± 0.2	0.1974 ± 0.0017	7.9 ± 0.8
KR 32	83	F	<3	9.4 ± 2.0	3.7 ± 2.5	2.05 ± 0.1	0.1903 ± 0.0017	4.7 ± 0.8
KR 33	78	F	<2	4.4 ± 1.4	<2	13.0 ± 4.3	0.2305 ± 0.0037	23.0 ± 1.7
KR 34	67	F	<1	11.1 ± 1.6	6.7 ± 1.5	7.8 ± 0.5	0.1976 ± 0.0015	8.0 ± 0.7
KR 35	78	F	<23	39.6 ± 12.3	<2	6.1 ± 0.4	0.1819 ± 0.0027	0.9 ± 1.2
KR 36	65	F	<2	21.4 ± 1.8	12.6 ± 2.4	9.2 ± 3.0	0.1904 ± 0.0019	4.7 ± 0.9
KR 37	76	F	<3	7.7 ± 1.6	<3	4.6 ± 0.4	0.1772 ± 0.0022	<1.0
KR 38	56	F	<3	10.6 ± 1.4	9.9 ± 3.4	3.6 ± 0.6	0.1760 ± 0.0040	<1.8
KR 39	66	M	<2	17.7 ± 2.4	6.1 ± 1.6	3.4 ± 0.4	0.1919 ± 0.0012	5.4 ± 0.5
KR 40	60	F	<10	7.0 ± 3.2	–	3.1 ± 0.3	0.1735 ± 0.0038	<1.7
KR 41	79	F	<6	36.6 ± 5.1	27.7 ± 11.7	3.7 ± 0.4	0.1999 ± 0.0019	9.0 ± 0.9
KR 42	77	M	<2	11.1 ± 1.6	10.6 ± 2.5	2.1 ± 0.1	0.1957 ± 0.0021	7.1 ± 1.0
KR 43	83	M	<5	26.0 ± 3.8	7.1 ± 5.1	3.0 ± 0.1	0.1965 ± 0.0016	7.5 ± 0.7
KR 44	73	F	<3	11.7 ± 2.1	5.1 ± 2.3	5.2 ± 0.2	0.1940 ± 0.0038	6.4 ± 1.7
KR 45	88	F	<4	10.6 ± 3.0	11.9 ± 4.6	17.8 ± 0.9	0.1948 ± 0.0031	6.7 ± 1.4
KR 46	87	F	<18	42.4 ± 11.9	17.9 ± 7.9	2.3 ± 0.5	0.1923 ± 0.0021	5.6 ± 1.0
KR 47	80	F	<8	17.8 ± 4.8	5.1 ± 4.6	3.1 ± 0.7	0.1988 ± 0.0079	8.5 ± 3.6
KR 48	56	F	<4	15.9 ± 3.2	15.0 ± 3.4	6.6 ± 0.7	0.1905 ± 0.0019	4.8 ± 0.9
KR 49	46	F	<5	2.3 ± 1.9	<5	2.7 ± 0.4	0.1541 ± 0.0077	–
KR 50	77	F	<12	27.4 ± 7.5	12.4 ± 4.2	2.2 ± 0.2	0.1689 ± 0.0027	–
KR 51	79	F	<2	11.2 ± 2.0	6.5 ± 2.5	2.9 ± 0.4	0.1747 ± 0.0020	<0.9
KR 52	84	F	<8	26.6 ± 6.9	10.1 ± 5.7	1.6 ± 0.3	0.1915 ± 0.0016	5.2 ± 0.7
KR 53	56	F	<5	20.9 ± 3.0	9.4 ± 4.1	2.3 ± 0.3	0.1978 ± 0.0013	8.1 ± 0.6
KR 54	81	F	<8	5.5 ± 2.8	<5	–	0.1784 ± 0.0014	<0.6
KR 55	69	M	<13	35.4 ± 6.8	<2	6.6 ± 0.8	0.1852 ± 0.0018	2.4 ± 0.8
KR 56	73	F	<8	9.6 ± 2.9	9.4 ± 5.4	4.1 ± 0.4	0.1888 ± 0.0016	4.0 ± 0.7
KR 57	49	F	<2	4.9 ± 1.0	4.3 ± 2.7	6.0 ± 1.8	0.1874 ± 0.0016	3.4 ± 0.7
KR 58	48	M	<4	3.2 ± 1.6	<5	–	0.1866 ± 0.0037	3.0 ± 1.7
KR 59	45	M	<5	2.9 ± 1.2	<5	9.9 ± 1.5	0.1473 ± 0.0036	–
KR 60	41	F	<6	<2	4.0 ± 3.4	3.3 ± 0.4	0.1582 ± 0.0035	–
BP 1	53	M	–	–	5.4 ± 2.6	1.8 ± 0.4	–	–
BP 2	88	F	–	–	9.4 ± 4.1	–	–	–
BP 3	74	F	<3	21.4 ± 3.2	8.8 ± 2.4	–	0.1933 ± 0.0013	6.0 ± 0.6
BP 4	80	F	<2	13.2 ± 2.1	5.1 ± 1.9	7.5 ± 2.7	0.1954 ± 0.0012	7.0 ± 0.5
BP 5	70	F	<4	21.1 ± 3.9	3.1 ± 2.8	2.7 ± 0.5	0.1925 ± 0.0020	5.7 ± 0.9
BP 6	56	M	<3	8.8 ± 2.0	4.0 ± 1.5	3.7 ± 0.8	0.1772 ± 0.0019	<0.9
BP 7	77	F	<3	14.1 ± 2.6	13.4 ± 2.6	3.4 ± 0.8	0.1929 ± 0.0013	5.9 ± 0.6
BP 8	71	F	<2	14.7 ± 2.3	5.0 ± 2.1	9.9 ± 4.3	0.1942 ± 0.0009	6.5 ± 0.4
BP 9	68	M	<4	17.3 ± 3.3	13.9 ± 2.7	4.8 ± 2.4	0.1963 ± 0.0016	7.4 ± 0.7
BP 10	66	M	<3	21.7 ± 3.0	8.9 ± 3.4	–	0.1954 ± 0.0014	7.0 ± 0.6
BP 11	59	F	<2	6.6 ± 1.7	2.1 ± 1.6	3.0 ± 1.7	0.1802 ± 0.0015	0.1 ± 0.7
BP 12	55	F	<3	7.2 ± 1.7	4.6 ± 2.6	4.9 ± 2.6	0.1764 ± 0.0012	<0.5
BP 13	56	M	<3	12.7 ± 2.4	4.6 ± 1.9	5.3 ± 2.8	0.1755 ± 0.0011	<0.5
BP 14	90	M	<2	22.6 ± 2.8	16.3 ± 6.1	9.0 ± 4.6	0.1830 ± 0.0012	1.4 ± 0.5
BP 15	76	F	<5	10.2 ± 2.7	<2	3.8 ± 2.0	0.1787 ± 0.0013	<0.6
BP 16	57	M	<2	15.1 ± 2.0	5.1 ± 1.8	5.1 ± 2.7	0.1140 ± 0.0006	–
BP 17	81	F	<4	22.9 ± 3.6	6.4 ± 3.7	8.1 ± 4.2	0.1837 ± 0.0011	1.7 ± 0.5
BP 18	81	F	<3	15.0 ± 2.9	6.1 ± 3.1	9.3 ± 4.9	0.1840 ± 0.0013	1.8 ± 0.6
BP 19	70	F	<2	12.3 ± 3.7	3.7 ± 2.5	4.5 ± 3.2	0.1903 ± 0.0014	4.7 ± 0.6
BP 20	64	M	<2	10.3 ± 1.7	11.2 ± 5.6	5.5 ± 4.0	0.1856 ± 0.0011	2.5 ± 0.5
BP 21	77	F	<2	26.6 ± 3.2	5.1 ± 3.7	2.4 ± 1.9	0.1892 ± 0.0006	4.2 ± 0.3
BP 22	55	F	<4	10.8 ± 2.5	<4	5.6 ± 4.0	0.1796 ± 0.0013	<0.6
BP 23	70	F	<2	12.3 ± 2.2	<4	5.2 ± 3.8	0.1856 ± 0.0018	2.5 ± 0.8
BP 24	32	M	<8	<2	<4	10.9 ± 16.3	0.1139 ± 0.0064	–
BP 25	68	F	<3	50.1 ± 3.9	8.6 ± 3.5	13.0 ± 1.4	0.0459 ± 0.0004	–
BP 26	64	M	<9	<2	5.8 ± 4.2	6.8 ± 1.0	0.1740 ± 0.0027	<1.2
BP 27	59	F	<6	7.8 ± 2.9	4.2 ± 4.1	19.3 ± 2.3	0.1801 ± 0.0104	<4.7
BP 28	85	F	<5	26.5 ± 4.7	20.5 ± 1.9	4.1 ± 0.4	0.1864 ± 0.0010	2.9 ± 0.5

**Fig. 2:**
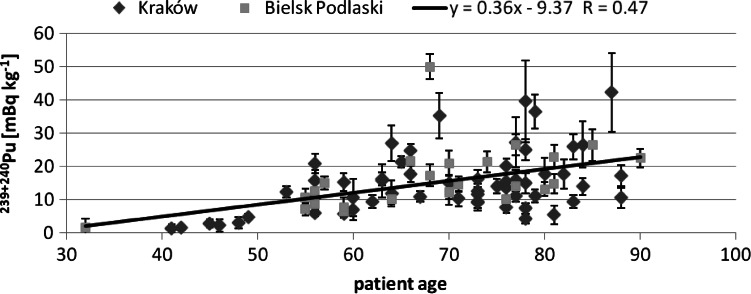
Patients’ age—^239+240^Pu concentration correlation

**Fig. 3:**
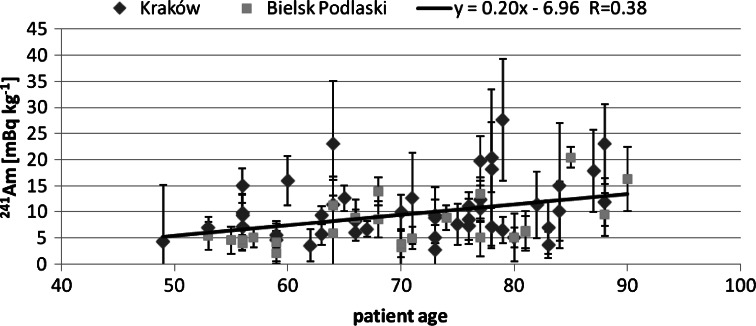
Patients’ age and ^241^Am concentration correlation

**Fig. 4:**
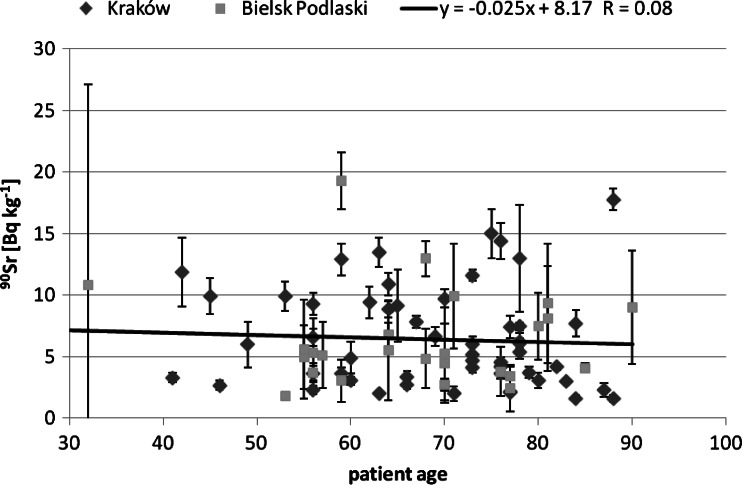
Patients’ age and ^90^Sr concentration correlation

**Fig. 5:**
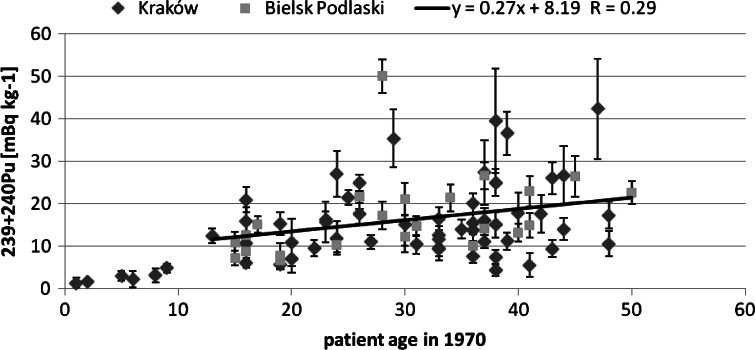
Correlation between the patients’s age at seventies and ^239+240^Pu concentration (patients younger than 10 years old in 1970 were excluded from the fit of the *trend line*)

Next, we tried to find correlation between the studied isotopes concentration and the patients age. The obtained correlation results are presented in Figs. 2, 3 and 4. For Pu and Am one can see an increasing trend, the older the person was, the higher activity Pu and Am concentration were found. This was observed already for a smaller group of 28 analysed human bone samples [13]. On the contrary, for Sr such a trend could not be observed. As the main pathway of Pu is inhalation, the increasing trend may suggest that the observed Pu was systematically accumulated over the patient‘s life. On the other hand, in Fig. 5 we can see a difference between a child patient at 1970 and the rest of the investigated people. It is generally known that after 1963 the USA, Soviet Union and UK stopped their atmospheric weapon tests, thus Pu concentration in air kept falling down smoothly over the next years by orders of magnitude. This difference can be explained by much less lungs air exchange efficiency in children. Moreover, the increase in the activity concentration with age can be also interpreted in terms of losing bones mass while aging—the bone mass is in the denominator and keeps diminishing. Therefore, the increasing trend is of solely spurious nature as the observed concentration had been accumulated mainly in the fifties and sixties of the last century, when the number of atmospheric tests reached its maximum.

**Fig. 6:**
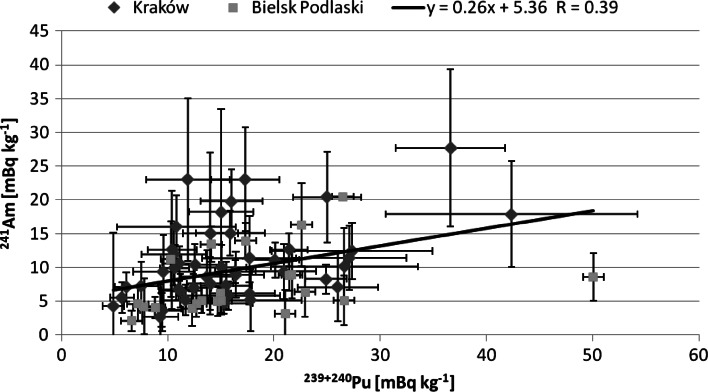
^241^Am and ^239+240^Pu concentration correlation

Correlation between Pu and Am concentration was also analysed (Fig. 6). The coefficient of trend line equal to 0.26 is very close to the model ratio between activity of ^241^Am and ^239+240^Pu for the global fallout, i.e. 0.33. This may suggest the global fallout to be the main source of contamination in examined humans.

**Fig. 7:**
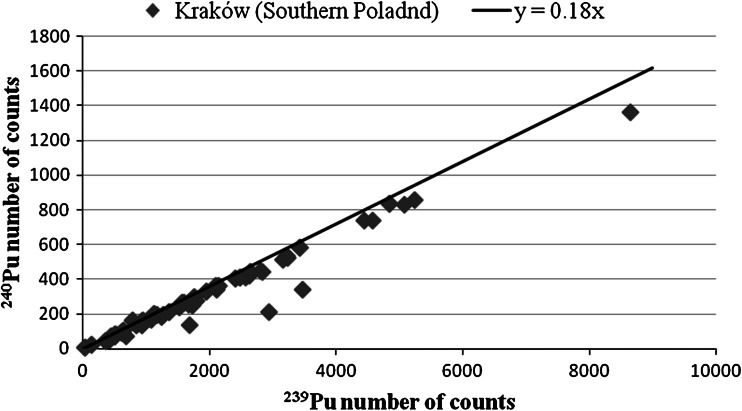
Pu mass ratio in Krakow (Southern Poland)

**Fig. 8:**
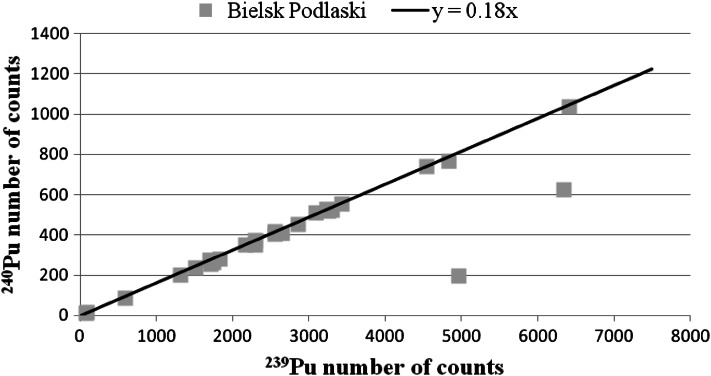
Pu mass ratio in Bielsk Podlaski (Northeastern Poland)

The mass spectrometry range obtained for Pu ratios in both populations are presented in Table 5. Table 6 shows the range of Chernobyl fraction. The mass ratios and the Chernobyl fraction for each sample are presented in Table 4. Pu mass ratio for each sample are also presented in Figs. 7 and 8. As we can see in both populations the majority of the samples display ratios characteristic for the global fallout. In several samples we also found very unusual relatively low ratios that may suggest nuclear weapon construction materials to have participated into it. We did not manage to formulate a good explanation to it. Chernobyl fraction in both population tends to be marginal. More than a half (56 %) of all the results showed Chernobyl fraction to be below 5 %. Only in two samples Chernobyl fraction above 10 % was detected.

**Table 5:** The range of Pu mass (^240^Pu/^239^Pu) ratios for both populations

Population	Min	Max	Average	Median
Kraków (Southern Poland)	0.1735 ± 0.0038	0.2305 ± 0.0035	0.1907 ± 0.0175	0.1911 ± 0.0015
Bielsk Podlaski (Northeastern Poland)	0.1740 ± 0.0027	0.1963 ± 0.0016	0.1856 ± 0.0143	0.1856 ± 0.0018

**Table 6:** Calculated percentage of Chernobyl Pu fraction for both populations

Population	Min	Max	Average	Median
Kraków (Southern Poland)	<0.9	23 ± 1.7	5.0 ± 3.7	5.0 ± 0.7
Bielsk Podlaski (Northereastern Poland)	<0.6	7.4 ± 0.7	2.9 ± 2.8	2.5 ± 0.8

The current results can be compared to the existing data on Pu content in human bones. Table 7 presents such a comparison for activity concentration range of bone-seeking isotopes obtained currently with a set of examples to the data already existing [13, 22–28]. Apart from cases where bones of accident victims were examined, the obtained results are very similar to the ones reported previously for a worldwide population.

**Table 7:** Comparison of the results obtained currently (the last row) for artificial bone seeking radioisotopes with similar studies conducted all over the world

Site	^239+240^Pu (mBq kg^−1^)	^241^Am (mBq kg^−1^)	^90^Sr (Bq kg^−1^)	Remarks	Reference
Marshall Isl.	(8 ± 2) ÷ (46 ± 4)	(8 ± 4) ÷ (19 ± 8)	–		[22]
Nepal	(10.0 ± 3.3) ÷ (73.3 ± 13.3)	–	–		[23]
Australia	<8.3 ÷ (23.3 ± 11.7)	–	–		[23]
Germany	–	–	(7.3 ± 2.2) ÷ (13.7 ± 4.1)	Tooth	[24]
Northwest Ukraine	–	–	(19.7 ± 5.9) ÷ (30.4 ± 9.1)	Tooth	[24]
Belgium	2.9 ÷ 20.2	–	–	Accident	[25]
U.S.A.	25 ÷ 36,036	14 ÷ 2,365,000	–	LANL (incl. accident)	[26]
Southern Poland	(5.7 ± 1.1) ÷ (27.1 ± 5.4)	(2.8 ± 2.4) ÷ (23.4 ± 12.2)	(1.6 ± 0.3) ÷ (15 ± 2)		[13]
Poland	<2 ÷ (50 ± 4)	<2 ÷ (35 ± 12)	(1.6 ± 0.3) ÷ (19 ± 2)		This work

A more complex statistical studies, such as correspondence analysis, were preformed to find any relationship between the observed activity concentration and certain properties, or individual habits reported in the questionnaire. The results were already published elsewhere, in the proceedings of a local conference [29], nevertheless it worth quoting that among different parameters used in correspondence analysis, the strongest relationship between the high levels of activity concentration of ^241^Am and ^90^Sr in bones, and intense cigarette smoking and declared high level mushrooms consumption was found. It is also worth highlighting that higher levels of activity concentration of ^241^Am and ^90^Sr in bones correlated with patients’ age range 61–73 year, which is in accordance with the above hypothesis that these patients incorporated the majority of radioactive contamination during their childhood, i.e. in fifties and sixties of the twentieth century.

## Conclusions

We could not see any differences between the studied populations. In both groups the level of internal contamination is comparable, and Chernobyl fraction (if any) seems to be marginal. It is, however, highly probable that the observed concentration for Pu and Am had been accumulated in the fifties and sixties of the last century when the number of nuclear atmospheric explosions had maximum. In both populations the Chernobyl fraction is usually well below 10 %.

The correlation between Pu and Am concentration in human bones, and the age suggested in our previous study [13] carried out on much smaller number of patients has been confirmed; similarly lack of such correlation for ^90^Sr was reported.

Since the examined people were general public and no local, Polish sources for Pu release into environment are available, our results can be treated as background values for Central European population.
